# FDA announces recall of over 300 000 arrow radial artery catheterization kits: a call for action – an editorial

**DOI:** 10.1097/MS9.0000000000002364

**Published:** 2024-07-12

**Authors:** Hritvik Jain, Jyoti Jain, Ramez M. Odat, Aman Goyal, Rukesh Yadav

**Affiliations:** a Department of Internal Medicine, All India Institute of Medical Sciences (AIIMS), Jodhpur; b Department of Internal Medicine, Seth GS Medical College and KEM Hospital, Mumbai, India; c Faculty of Medicine, Jordan University of Science and Technology, Irbid, Jordan; d Department of Internal Medicine, Maharajgunj Medical Campus, Institute of Medicine, Tribhuvan University, Nepal

On 4 April 2024, the Food and Drug Administration (FDA) identified ARROW QuickFlash Radial Artery and Radial Artery/Arterial Line Catheterization Kits as causing serious adverse events and even death in over 194 registered complaints. Hence, the FDA issued a warning to recall these catheterization kits and avoid their further usage, considering this as a ‘Class I recall’, the most serious type of recall^1^. Radial artery catheterization is a commonly performed intervention in critical cases for intensive hemodynamic monitoring and vasopressor administration, particularly in cases of shock. Arterial blood catheters also provide direct access for frequent blood gas analyses in patients on ventilatory support for continuous monitoring. Venous catheters are also frequently used; however, their limitations include an increased risk of infection, bleeding, venous obstruction, cardiac tamponade, emboli, and sepsis^2^. Although arterial lines are also associated with the aforementioned adverse events, they offer a much more accurate measurement of blood pressure^3^. Arterial blood pressure wave analysis is critical in making informed decisions, particularly in severely hypotensive patients on external life support^4^. Despite its importance, the failure rates of radial arterial catheters are greater than 25% and include complications such as loss of catheter function, absence of blood return, dislodgement, and unanticipated stoppage of hemodynamic monitoring^5^.

The Arrow QuickFlash Arterial Catheter is a type of arterial catheter composed of integrated wings, hydrophilic coating, flashback windows, blood containment, and a removable guidewire. The Arrow QuickFlash catheter was marketed under the impression that it allowed for the rapid identification of the return of blood and reduced arterial transfixation. Teleflex claimed the Arrow QuickFlash arterial catheter to have quick handling (easy insertion with one hand), quick visualization, quick safety procedures (minimizing blood exposure), and quick alternative techniques using guidewire repositioning^6^. However, Teleflex received over 190 reported complaints concerning increased resistance in the guidewire handle, which obstructed its free use during the intervention. These complaints were reports of adverse events such as injury to the arterial vessel walls, vasospasm, embolism, and death. In contrast, other brands of arterial catheters have their own sets of complications but typically report fewer complaints of such severe outcomes. For example, while complications such as catheter dislodgement and infection are common across other brands such as RadiaFlo and McKesson, the specific problem of guidewire resistance leading to severe vascular injury and embolism appears more frequently in Arrow QuickFlash catheters. On 12 February 2024, Teleflex and Arrow issued an Urgent Recall letter to hospitals and distributors to refrain from using ARROW QuickFlash Radial Artery and Radial Artery/Arterial Line Catheterization Kits to avoid serious outcomes among recipients of these devices. FDA recommended quarantining such catheterization kits to avoid mistaken usage^1^.

This current editorial highlights the pitfalls of Arrow catheterization kits and is a call to action that warns cardiovascular clinicians to refrain from cannulating patients with the aforementioned catheter.

## Ethical approval

The information provided in the manuscript does not require an ethics application or approval.

## Consent

No patients were sorted. Informed consent was not required for this manuscript.

## Source of funding

No funding was received.

## Author contribution

H.J.: conceptualization, methodology, supervision, and writing – review and editing; J.J.: supervision, investigation, writing – original draft, and writing – review and editing; R.M.O.: resources, project administration, writing – original draft, and writing – review and editing; A.G.: validation, writing – original draft, and writing – review and editing; R.Y.: validation, writing – original draft, and writing – review and editing.

## Conflicts of interest disclosure

The authors declare no conflicts of interest.

## Research registration unique identifying number (UIN)

Not applicable.

## Guarantor

Hritvik Jain.

## Data availability statement

Available through the manuscript. No other data were generated.

## Provenance and peer review

Not applicable.
